# Rapamycin Enhances the Anti-Cancer Effect of Dasatinib by Suppressing Src/PI3K/mTOR Pathway in NSCLC Cells

**DOI:** 10.1371/journal.pone.0129663

**Published:** 2015-06-10

**Authors:** Bin Chen, Xin Xu, Jie Luo, Heyong Wang, Songwen Zhou

**Affiliations:** 1 Department of Medical Oncology, Shanghai Pulmonary Hospital, Shanghai, China; 2 School of Medicine Cancer Institute, Tongji University, Shanghai, China; Emory University, UNITED STATES

## Abstract

Src and the mammalian target of rapamycin (mTOR) signaling are commonly activated in non-small cell lung cancer (NSCLC) and hence potential targets for chemotherapy. Although the combined use of Src inhibitor Dasatinib with other chemotherapeutic agents has shown superior efficacy for cancer treatment, the mechanisms that lead to enhanced sensitivity of Dasatinib are not completely understood. In this study, we found that Rapamycin dramatically enhanced Dasatinib-induced cell growth inhibition and cell cycle G1 arrest in human lung adenocarcinoma A549 cells without affecting apoptosis. The synergistic effects were consistently correlated with the up-regulation of cyclin-dependent kinases inhibitor proteins, including p16, p19, p21, and p27, as well as the repression of Cdk4 expression and nuclear translocation. Mechanistic investigations demonstrated that FoxO1/FoxO3a and p70S6K/4E-BP1, the molecules at downstream of Src-PI3K-Akt and mTOR signaling, were significantly suppressed by the combined use of Dasatinib and Rapamycin. Restraining Src and mTOR with small interfering RNA in A549 cells further confirmed that the Src/PI3K/mTOR Pathway played a crucial role in enhancing the anticancer effect of Dasatinib. In addition, this finding was also validated by a series of assays using another two NSCLC cell lines, NCI-H1706 and NCI-H460. Conclusively, our results suggested that the combinatory application of Src and mTOR inhibitors might be a promising therapeutic strategy for NSCLC treatment.

## Introduction

Non-small cell lung cancer (NSCLC) is the major pathological subtype of lung cancer which is the most common cause of death from cancer worldwide [1]. Among NSCLC patients, the Src family kinases (SFKs) are constitutively overexpressed or activated [2,3]. As a potential therapeutic target for NSCLC, Src might play an important role in the progression of lung adenocarcinomas via regulating signals from multiple cell surface molecules, including integrin, growth factors, and G protein coupled receptors [4,5]. Preclinical studies have shown that SFKs inhibition can suppress proliferation, angiogenesis, invasion, and survival of cancer cells [6–9]. As the specific inhibitor of Src, Dasatinib has been approved for the treatment of chronic myeloid leukemia (CML), and it is now being evaluated for the clinical use in lung cancer [10,11]. However, Dasatinib as monotherapy exhibited modest clinical activity that was lower than that generally observed in NSCLC patients who received chemotherapy [11]. In contrast, the combination of Dasatinib with cytotoxic chemotherapy appeared more promising than using as a single agent. Since Src can mediate tumor resistance to cytotoxic chemotherapy, Src inhibition by Dasatinib has been demonstrated to enhance the response of colon and lung cancer cells to cisplatin in vitro [12,13]. In addition, a recent clinical trial of Dasatinib in combination with erlotinib achieved enhanced beneficial effect of the treatment in patients with previously treated NSCLC [14]. Moreover, Dasatinib could also facilitate the anticancer effects of radiotherapy [15]. Although the superior efficacy has strongly suggested that combination with Dasatinib is of critical importance for NSCLC therapies, the mechanisms that lead to enhanced sensitivity of chemotherapies are still complex and not fully understood. Given that Src modulates signal transductions governing proliferation, invasion, apoptosis, *etc*. of cancer cells, studies deciphering the regulation of Src activation and its interaction with other signaling molecules in cancer therapy are particularly warranted.

Besides Src, the mammalian target of rapamycin (mTOR) is also highly activated in many lung cancer patients and represents as another target for therapy. The mTOR signaling pathway drives many major cellular processes and is implicated in an increasing number of pathological conditions including cancer [16]. Recently, preliminary clinical data have indicated a certain antitumor activity of the mTOR inhibitor Rapamycin and its analogues in some cancers including NSCLC [17,18]. Notably, Rapamycin is also being explored for its ability to restore sensitivity of cancer cells to upstream signaling targeted agents [19]. Akt/mTOR inhibition by Rapamycin or its derivatives have appeared to synergistically enhance the cytotoxicity of radiation and chemotherapeutic agents, promoting the induction of cell cycle arrest and apoptosis [20,21]. On the other hand, it has been shown that mTOR could be activated by Src signaling through phosphatidylinositol 3-kinase (PI3K)/Akt pathway [22–24]. This led to the hypothesis that the combination of Rapamycin with Src inhibitors, such as Dasatinib, could facilitate the anticancer activity and be more efficacious in chemotherapy. A recent study demonstrated that dual inhibition of Src and mTOR was highly effective on tumor regression in mouse models of breast cancer [25]. However, no preclinical data are currently available with their combinatory use for the treatment of lung cancer. Also, the potential effect of mTOR in the regulation of Src signaling remains largely unclear.

Here, we aim to investigate the combinatory therapeutic effect of Rapamycin and Dasatinib on lung cancer by using human lung adenocarcinoma cells (A549), lung squamous carcinoma cells (NCI-H1703) and large-cell lung cancer cells (NCI-H460) as in vitro cellular models. In this study, Dasatinib-induced cell growth inhibition and cell cycle arrest were dramatically enhanced by the co-treatment with Rapamycin, paralleling with then up-regulation of the cyclin-dependent kinases (CDKs) inhibitor proteins. Analysis on the signaling molecules showed that Src deactivation was efficiently facilitated by mTOR inhibition via PI3K-Akt pathway. Using small interfering RNAs against Src and mTOR, we observed similar repression to those from the inhibitors on cell proliferation, cell cycle progression, invasion, and migration in A549 cells. Furthermore, our results revealed the synergistic interaction between Src and mTOR signalings in NSCLC cells, which suggested the promising therapeutic benefit of mTOR/Src dual inhibition for NSCLC treatment.

## Materials and Methods

### Reagents and antibodies

Dasatinib and Rapamycin were purchased from Selleck Chemical (Shanghai, China). Primary antibodies against phospho-Src (Tyr416), Src, phospho-mTOR (Ser2448), mTOR, phospho-PI3K (Tyr458), PI3K p85, phospho-Akt (Thr308), Akt, phospho-FoxO1 (Ser256), phospho-FoxO3a (Ser253), phospho-4E-BP1 (Thr37/46), 4E-BP1, Cdk2, Cdk4, Cdk6, and Alexa Fluor 555 conjugated antibody were obtained from Cell Signal Technologies (Shanghai, China). Primary antibodies against CDK inhibitor proteins including p16, p19, p21, p27, and β-tubulin were purchased from Santa Cruz Biotechnology (Shanghai, China). Antibodies against phospho-p70S6K (Thr389/412), p70S6K, were purchased from SAB Signalway (College Park, MD, USA). The Secondary HRP- or FITC- conjugated antibodies, si-mTOR, si-Src, and transfection reagents were purchased from Santa Cruz Biotechnology (Shanghai, China).

### Cell culture and treatment

The human adenocarcinoma cell line A549, human lung squamous carcinoma cell line NCI-H1703, human large-cell lung cancer cell line NCI-H460 and normal bronchial epithelial cell BEAS-2B were purchased from ATCC (Beijing, China), and cultured in dulbecco's modified eagle medium (Life technology, Shanghai, China) supplemented with 10% fetal bovine serum, at 37°C in a humidified atmosphere with 5% CO_2_. Cell number and viability were determined by trypan blue exclusion, with at least 90% viable cells before treatment exposure. A549 cells were treated with Dasatinib (5, 10, 25, or 50 nM) alone or in combination with Rapamycin (20, 50, or 100 nM) for 24 to 96 hours. Cells treated with 0.1% dimethylsulfoxide (DMSO) were used as vehicle control.

### Cell proliferation and cell cycle assays

For cell proliferation assay, 10^4^ cells per well were seeded in 24-well plates and incubated for 24 h. The cells were treated with 5, 10, 15, or 20 nM of Dasatinib in the presence or absence of Rapamycin (20, 50, or 100 nM), and harvested at 24, 48, 72, and 96 h. Trypan blue exclusion assays were carried out to determine cell numbers using a Cellometer Auto T4 (Nexcelom Bioscience, Lawrence, MA, USA).

For cell cycle assay, cells in 6-well plates were collected, rinsed and fixed overnight in 75% cold ethanol at -20°C. Then, cells were treated with Tris-HCl buffer (pH 7.4) with 100 μg/mL RNase A and stained with 25 μg/mL propidium iodide (PI) (Life technology, Shanghai, China). Ten thousands cells were acquired and analyzed by flow cytometry (BD FACSCalibur, CA, USA) based on DNA content.

### Apoptosis assay

1×10^6^ cells were treated with DMSO (0.1%), Dasatinib (10 nM) alone or in combination with Rapamycin (100 nM) for 96 h. Cell apoptosis rate was determined by Annexin V-FITC Apoptosis Detection Kit I (BD Biosciences, Shanghai, China) according to the manufacturer’s instruction. Fluorescent intensities of all probes were determined by flow cytometry (BD FACSCalibur, CA, USA).

### Real-time quantitative PCR

Total RNA was isolated using Trizol reagent and treated with DNase according to the manufacturers’ instructions. First-strand cDNA was synthesized using M-MLV reverse transcriptase and oligo-dT primer (Invitrogen, Shanghai, China). Quantitative real-time PCR assays were performed in 96-well optical plates on an ABI Prism 7000 Sequence Detection System (Lifetechnology, Shanghai, China) with SYBR Green qPCR Master Mix (Qiagen, Shanghai, China). Expression of P16, P19, P21, P27, Cyclin A/D1/E and CDC25A mRNA was normalized against that of 18s RNA. Primer sequences for real-time PCR assays were listed in S1 Table.

### Western blotting

Homogenate proteins (20 μg) from each treated cells were resolved on SDS-polyacrylamide gels, and electrophoretically transferred onto Immobilon-P polyvinylidenedifluoride membranes. Subsequently, membranes were incubated with specific antibodies against CDK inhibitor proteins (p16, p19, p21 and p27), cdk2/4/6, Forkhead box proteins (FoxO1 and FoxO3a), the phosphorylated or total Src, PI3K, Akt, mTOR, p70S6K and 4E-BP1, respectively. Beta-tubulin was used for normalization of protein loading. After incubating with horseradish peroxidase (HRP)-conjugated IgG antibody, membranes were developed using ECL Western blotting detection reagent (Thermo, Beijing, China). Densitometric measurements of the bands were performed using Quantity One software program (Bio-Rad, Beijing, China).

### Cell immunofluorescence staining

After seeded in 12-well plate for 24 h, A549 cells were treated with DMSO (0.1%), Dasatinib (10 nM) alone or in combination with Rapamycin (100 nM) for 48 h. Cells were washed and fixed with 0.4% paraformaldehyde for 15 min at room temperature, then incubated with primary antibodies against phosphorylated Src or Cdk4 overnight at 4°C. After washing, the cells were incubated with FITC-labeled or Alexa Fluor 555 Conjugated secondary antibody. Subsequently, the cell nucleus were stained with PI and then observed using the Cytation 3 multiple imaging system (BioTek, VT, USA).

### Small interfering RNA (siRNA) transfection

A549 cells were seeded in a six well tissue culture plate by 2 x 10^5^ cells per well in 2 ml antibiotic-free medium and incubated for 24 h. According to the manufacture’s instruction, cells were incubated with transfection medium containing si-Src, si-mTOR, or control siRNA for 8 h. Then the transfection medium was replaced by normal fresh complete medium, followed by an additional incubation for 24 to 72 h. Subsequently, the cells were collected for assays on cell proliferation, cell cycle and western blotting, respectively.

### Cell invasion assay

Cell invasion assay was performed using trans-well chambers (Matrigel-coated membrane for invasion, BD Biosciences, Shanghai, China). A total of 5×10^5^ cells were plated in the upper chamber with serum-free medium and treated with 0.1% DMSO, Dasatinib (10 nM) alone or in combination with Rapamycin (100 nM). Then, the cells were allowed to invade toward 10% FBS as a chemoattractant in the lower chamber for 24h. Cells in the upper chamber were carefully removed using cotton swab, and cells on the bottom membrane were fixed and stained with nuclear fast red. The numbers of invaded cells were counted under microscope and the percent invasion was calculated by cells invading through the Matrigel matrix and membrane relative to the invasion of cells in the control group.

### Wound healing assay

Cells were seeded into 24-well tissue culture plates, and reached 70–80% confluence as a monolayer after 24h of growth. The monolayer was gently and slowly scratched with a new 1 ml pipette tip across the center of the well. Then well was wash twice with medium to remove detached cells. After replenishing the well with fresh medium, the cells were treated and grown for additional 18 h. The monolayer cells were photographed under an inverted phase contrast microscope.

### Statistical analysis

All data represent at least three independent experiments and are expressed as the mean ± standard deviation (SD) Statistical comparisons were performed using a software package SPSS 16.0 and analyzed with one-way analysis of variance (ANOVA) followed by a post hoc Dunnett’s test where appropriate. The statistical significance was set at p values < 0.05 (*), or < 0.01 (**).

## Results

### Rapamycin enhanced the inhibiting effect of Dasatinib on cell proliferation and cell cycle progression in A549 cells

**Fig 1:**
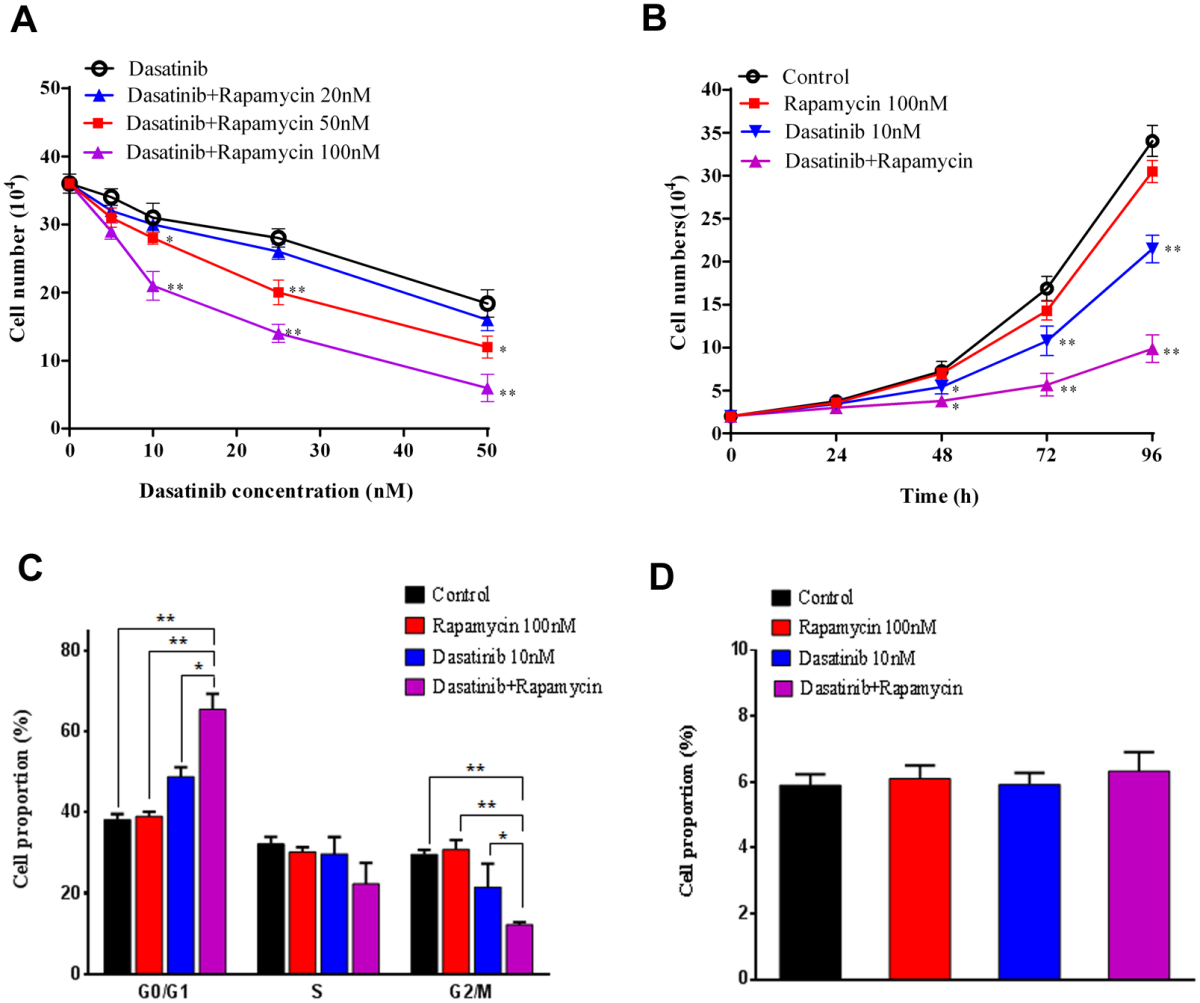
Rapamycin enhanced the inhibiting effect of Dasatinib on cell proliferation and cell cycle progression in A549 cells. (A) The concentration-dependent effect of Rapamycin on the anticancer activity of Dasatinib in A549 cells. As detailed in the “*Methods*”, A549 cells were treated with vehicle control (0.1% DMSO) or Dasatinib (5, 10, 25, and 50 nM) in the presence and absence of Rapamycin (20, 50, and 100 nM). Viable cell numbers were analyzed at 72 h after the co-treatment. (B) Effects of Rapamycin on the temporal changes of Dasatinib-induced growth inhibition in A549 cells. Cells were treated with vehicle control (0.1% DMSO), Dasatinib (10 nM) with or without Rapamycin (100 nM). Cell numbers were measured at 0, 24, 48, 72, and 96 h after the treatment. (C) Effects of Dasatinib and Rapamycin on the cell cycle distribution. A549 cells were treated with Dasatinib (10 nM) or Rapamycin (100 nM) for 96 h and analyzed by flow cytometry after PI staining. Results are expressed as the average percentage of cells at G0/G1, S, and G2/M phase from three independent experiments. (D) Effects of Dasatinib and Rapamycin on the apoptosis in A549 cells. Cells were treated with Dasatinib (10 nM) or Rapamycin (100 nM) for 96 h and the apoptotic rates were determined by flow cytometry with Annexin-V and PI staining. Columns, mean of three determinations; bars, SD. * p < 0.05, ** p < 0.01.

As shown in Fig 1A, Dasatinib at pharmacologically relevant levels markedly decreased the cell number of A549 cells in a concentration-dependent manner. Remarkably, the co-treatment with Rapamycin (50 and 100 nM) significantly enhanced Dasatinib-mediated growth inhibition in A549 cells, in that 10 nM of Dasatinib plus 100 nM of Rapamycin could achieve equal magnitudes of anticancer activity than Dasatinib alone at the concentration of 50 nM. Results about the temporal effects of Dasatinib with Rapamycin indicated that the A549 cell proliferation was significantly inhibited by Dasatinib (10 nM) through 96 h of treatment (Fig 1B). Importantly, the co-treatment with Rapamycin (100 nM) further decreased the cell numbers at as early as 48 h, suggesting the marked synergistic efficacy to the treatment of Dasatinib itself. However, Rapamycin alone showed minor suppression on the growth curve of cancer cells.

Previous studies have demonstrated that both Src and mTOR are often constitutively activated in acute myeloid leukemia (AML) cells and hence constitute potential therapeutic targets. For example, the epidermal growth factor receptor (EGFR) inhibitor erlotinib could induce cell cycle arrest at G1 phase by the inhibition of oncogenic signaling via Src and mTOR [26]. In agreement, our results from cell cycle assay in A549 cells showed that Dasatinib (10 nM) significantly increased the proportion of cells at G1 phase in comparison with the control group (Fig 1C), suggesting the direct arrest of cell cycle progression by Src inhibition in cancer cells. In contrast, Rapamycin (100 nM) alone exhibited little effect on the distribution of cells in various cell cycle phases. However, the presence of Rapamycin in the co-treatment with Dasatinib markedly increased the cell percentage at G1 phase, resulting in significant blockage of G1/S transition and cell mitosis. The result was consistent with the synergized inhibition of cell growth by Dasatinib and Rapamycin in combination. Meanwhile, the impact of Dasatinib and Rapamycin on cell apoptosis was also evaluated in A549 cells, but no significant difference of cell apoptosis rate was observed among treatments with control, Dasatinib or Rapamycin (Fig 1D), suggesting that Rapamycin and Dasatinib had minor effect on the apoptosis in A549 cells.

### Rapamycin potentiated Dasatinib to up-regulate CDK inhibitor proteins and down-regulate Cdk4

**Fig 2:**
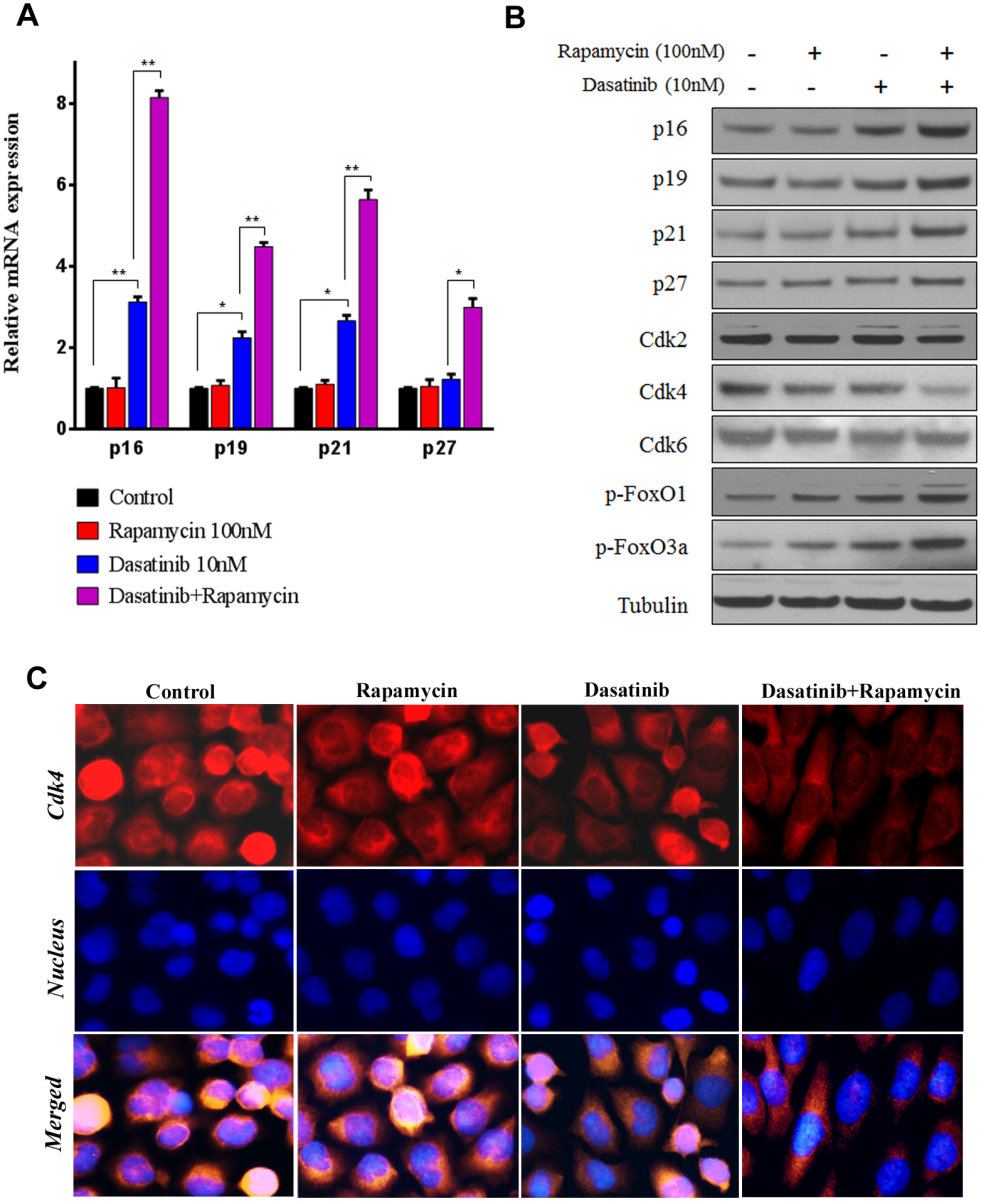
Rapamycin potentiated Dasatinib to up-regulate CDK inhibitor proteins and thereby down-regulate Cdk4. A549 cells were treated with vehicle control (0.1% DMSO) or Dasatinib (10 nM) in the presence and absence of Rapamycin (100 nM) for 24 h. (A) Relative expression of CDK inhibitor proteins (p16, p19, p21 and p27) at mRNA level. Columns, mean of three determinations; bars, SD. * p < 0.05, ** p < 0.01. (B) Expression of CDK inhibitor proteins, CDKs and FoxOs determined by western blotting. (D) Localization of Cdk4 determined by immunofluorescence staining. Representative pictures indicated staining of Cdk4 (red), nucleus (blue), and the merged images.

G1 cell cycle arrest is controlled by multiple factors including cyclins, cell division cycle protein (Cdc), CDKs and CDK inhibitor proteins. Our preliminary study demonstrated that the expressions of Cyclin A/D1/E and Cdc25A were not significantly changed by Dasatinib and Rapamycin at mRNA and protein levels (S1 Fig). As for CDK inhibitors, Dasatinib increased the expression of p16, p19, and p21 at both mRNA (Fig 2A) and protein (Fig 2B) levels, with statistical difference from the control groups. Interestingly, the co-treatment with Rapamycin markedly promoted Dasatinib-induced up-regulation of p16, p19, p21, and p27, but Rapamycin alone appeared to have little impact on the expression of these CDK inhibitor proteins. As the consequence, the expression of Cdk4 was dramatically suppressed by the combination of Dasatinib and Rapamycin, but neither Cdk2 nor Cdk6 seemed clearly responsive to the treatments (Fig 2B).

To further confirm the effects of Dasatinib and Rapamycin on CDK inhibitor proteins, the expression and distribution of Cdk4 in A549 cells was further analyzed by immunofluorescence staining. Normally, Cdk4 binds to cyclin D to form a complex in the cytoplasm, then accumulates on the nuclear membrane and translocate into the nucleus when cells progress through G1/S phase. Our result showed that Cdk4 predominantly localized in the nucleus in A549 cells; however, they were partly sequestered in the cytoplasm under the treatment with Dasatinib (Fig 2C). Furthermore, the translocation of Cdk4 into nucleus was significantly blocked by the treatment with Dasatinib and Rapamycin in combination. Taken together, these results consistently suggested that the inhibition of cell cycle progression by Dasatinib and Rapamycin was mediated through the up-regulation of CDK inhibitor proteins as well as the down-regulation of Cdk4 in A549 cells.

Previous reports have demonstrated that p19, p21 and p27 were transcriptionally regulated by FOXOs, which has later been proved as downstream targets of PI3K/AKT [25,27]. It was recently reported that p16 can be also regulated by Src-AKT pathway in cellular senescence in human prostate cancer cells [28]. In this study, our result indicated that the phosphorylation levels of FOXO1 and FOXO3a were also markedly increased by the co-treatment when compared to that induced by either Src or mTOR inhibitor alone (Fig 2B). Moreover, the result was in parallel with the expression of CDK inhibitors. Therefore, we speculated that the up-regulation of CDK inhibitors by Dasatinib and Rapamycin was probably attributed to the Src and PI3K/AKT pathway.

### Rapamycin synergized with Dasatinib in suppressing PI3K-Akt-mTOR signaling in A549 cells

**Fig 3:**
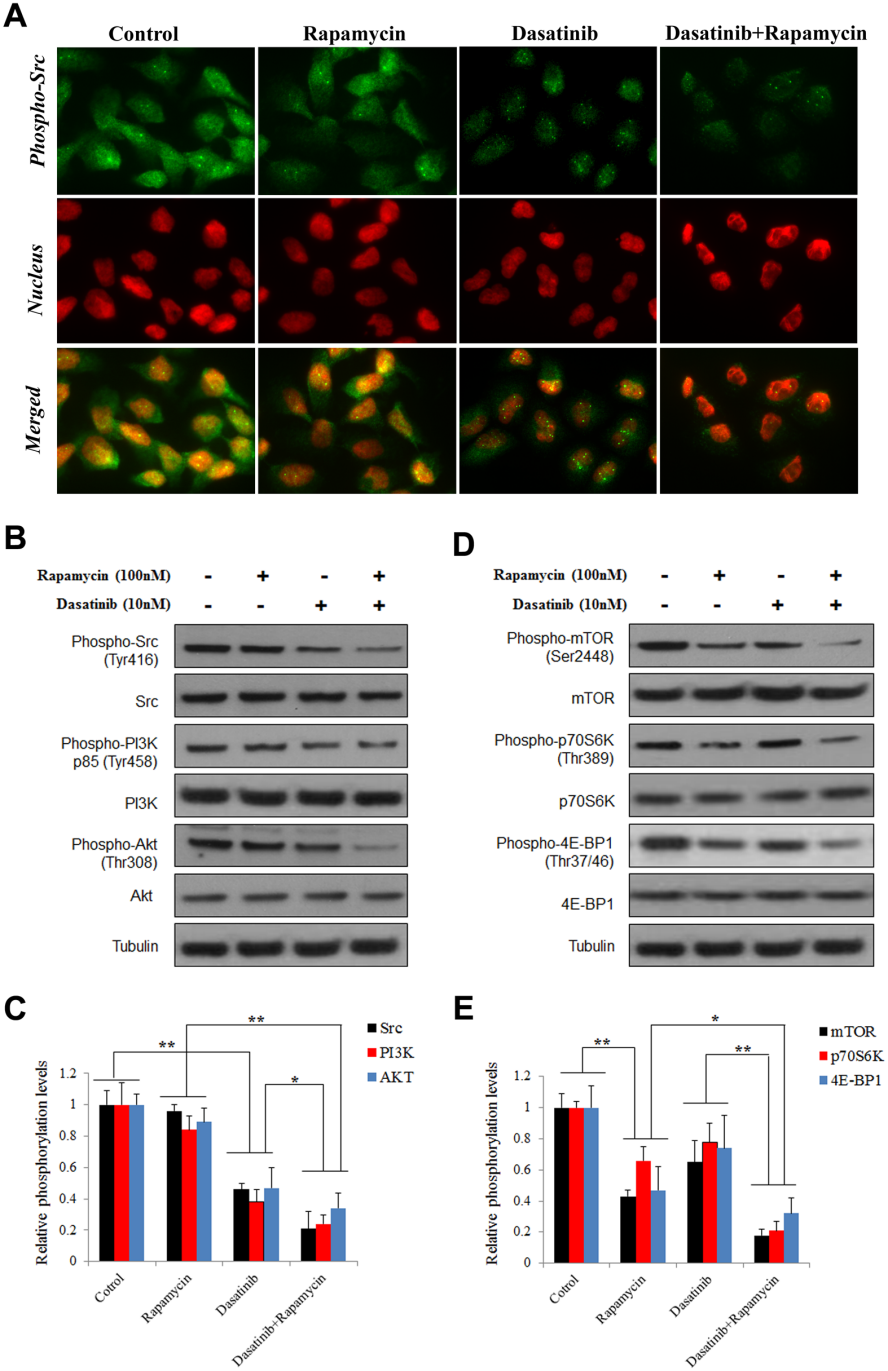
Dasatinib synergized with Rapamycin in the inhibition of Src/PI3K/Akt/mTOR signaling in A549 cells. A549 cells were treated with vehicle control (0.1% DMSO), Dasatinib (10 nM) with or without Rapamycin (100 nM) for 24 h. Homogenate proteins (20 μg) from the whole cell lysate was collected and used for western blotting. (A) Activation of Src determined by immunofluorescence staining. Representative pictures indicated staining of Src (red), nucleus (blue), and the merged images. (B) The activation of Src, PI3K, and Akt determined by western blotting. (C) Densitometry quantification of Src, PI3K and Akt phosphorylation in A549 cells. (D) The activation of mTOR signaling determined by western blotting. (E) Densitometry quantification of mTOR, p70S6K and 4E-BP1 phosphorylation in A549 cells. The data presented average relative phosphorylation ratios to the untreated control from three independent experiments. Columns, mean of three determinations; bars, SD. * p < 0.05, ** p < 0.01.

The most recent study demonstrated that Src phosphorylation may facilitate the activation of Akt-mTOR signaling pathway in AML cells [29]. To investigate the potential interaction between Src and mTOR pathways, we analyzed the activation of Src, PI3K, AKT, and mTOR in sequence. Firstly, the immunofluorescence staining of phospho-Src indicated that Src activation was inhibited by Dasatinib and more significantly repressed by the co-treatment with Rapamycin (Fig 3A), suggesting that mTOR inhibition by Rapamycin could synergize with Dasatinib in suppressing Src activation in A549 cells. And this result was further confirmed by western blotting (Fig 3B). In addition, our data demonstrated that the inhibition of Src by Dasatinib could also suppress the activation of PI3K-Akt signaling in A549 cells, which was in agreement with previous reports [29]. More importantly, the inhibitions of Src, PI3K, and AKT were unanimously enhanced by the presence of Rapamycin in comparison with Dasatinib alone, as shown in Fig 3B and 3C. It was worth noting that the result was well correlated with the observations regarding expression of CDK inhibitor and Forkhead box proteins (Fig 2B), which agreed with our speculation on the connection between cell cycle arrest and Src-PI3K-AKT deactivation. On the other hand, the immunostaining and western blotting results indicated that Src activation was inhibited by Rapamycin, to a less extent than Dasatinib. These data implied that mTOR which was conventionally recognized as the responsive target of PI3K/AKT signaling, might also play an important role in the regulation of Src activation.

Subsequently, the activation of mTOR was determined using western blotting on the phosphorylated levels of mTOR. As expected, the activation of mTOR was significantly inhibited by the inhibitor Rapamycin. Though Dasatinib exhibited moderate effect in repressing mTOR activation, the combination with Rapamycin remarkably advanced the inhibition that induced by Dasatinib alone (Fig 3D and 3E). Moreover, the phosphorylation of p70S6K and 4E-BP1, the known targets at the downstream of mTOR pathway, were both significantly suppressed by Rapamycin and they were also further decreased by the combination with Dasatinib. Collectively, these results suggested that Rapamycin synergized with Dasatinib in the repression of PI3K-Akt-mTOR signaling pathway in A549 cells.

### Dual repression of mTOR and Src by siRNAs induced cell cycle arrest and growth inhibition in A549 cells

**Fig 4:**
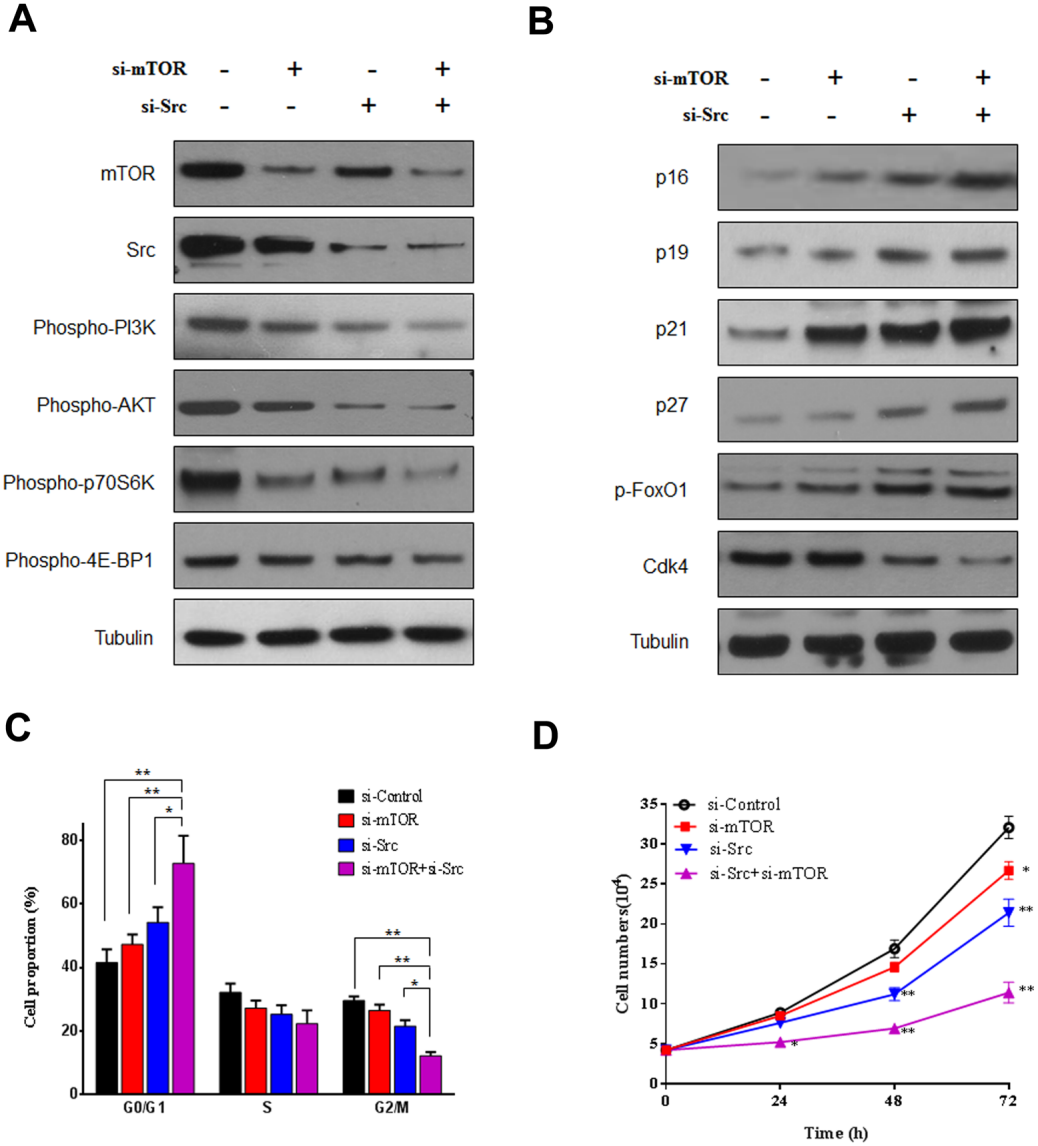
Repression of mTOR and Src by siRNAs facilitated cell cycle arrest and growth inhibition in A549 cells. (A) The activation levels of PI3K, AKT, and mTOR determined by western blotting. A549 cells were transfected with si-Src, si-mTOR, or control siRNA for 8 h, followed by a prolonged incubation of 24 h. Homogenate proteins (20 μg) from the whole cell lysate was collected and used for western blotting. (B) The expression of CDK inhibitor proteins (p16, p19, p21, and p27), FoxO1, and Cdk4 determined by western blotting. (C) Effects of si-mTOR and si-Src on the cell cycle progression. A549 cells were transfected with si-Src, si-mTOR, or control siRNA for 8 h, followed by an prolonged incubation of 24 h. Then the cells were collected and analyzed by flow cytometry with PI staining. Results are expressed as the average percentage of cells at G0/G1, S, and G2/M phase from three independent experiments. Results were expressed as the average percentage of cells at G0/G1, S, and G2/M phase from three independent experiments. (D) Effects of si-mTOR and si-Src on the temporal changes of cell proliferation. A549 ells were transfected with si-Src, si-mTOR, or control siRNA for 8 h, and then incubated with normal medium for 72 h. Cell numbers were measured at 0, 24, 48, and 72 h after the transfection. The data was presented as mean ± SD from three independent experiments. * p < 0.05, ** p < 0.01.

To validate the potential interaction between Src and mTOR signaling, we further investigated the alteration of phosphorylation levels of PI3K/AKT, and the expression levels of CDK inhibitor proteins in A549 cells with the artificial knock-down of Src or mTOR. As shown in Fig 4A, the restraining expression of Src or mTOR by siRNAs significantly repressed the phosphorylation levels of PI3K/AKT and p70S6K/4E-BP1, respectively. Conversely, the expression levels of the CDK inhibitor proteins, including p16, p19, p21, and p27, were all apparently elevated by the si-mTOR or si-Src (Fig 4B). In agreement, the expression of FoxO1 was also increased whereas Cdk4 significantly decreased. More importantly, the dual knock-down of mTOR and Src significantly promoted the inhibition of PI3K/AKT, p70S6K/4E-BP1, and resulted in the up-regulation of CDK inhibitor proteins, when compared to either si-mTOR or si-Src alone. The results were consistent with our findings in A549 cells treated with Rapamycin and Dasatinib.

In addition, the knock-down of mTOR and Src resulted in similar cell cycle arrest to that induced by the specific inhibitors Rapamycin and Dasatinib. As shown in Fig 4C, the dual inhibition of mTOR and Src by siRNAs induced more significant suppression of the cell cycle progression at G1 phase in A549 cells in comparison with either si-mTOR or si-Src alone. Moreover, the cell proliferation was also affected in the comparable manner with that induced by the chemical inhibitors (Fig 4D). It was notable that si-mTOR alone also induced moderate G1 arrest and growth inhibition in A549 cells, possibly due to its higher efficacy in depressing mTOR than that imposed by Rapamycin. Collectively, si-mTOR significantly enhanced the cell cycle arrest and growth inhibition that induced by si-Src in A549 cells, which supported our finding concerning the enhanced chemotherapeutic effect of Dasatinib in combination with Rapamycin.

### Rapamycin enhanced the inhibiting effect of Dasatinib on cell invasion and migration in A549 cells

**Fig 5:**
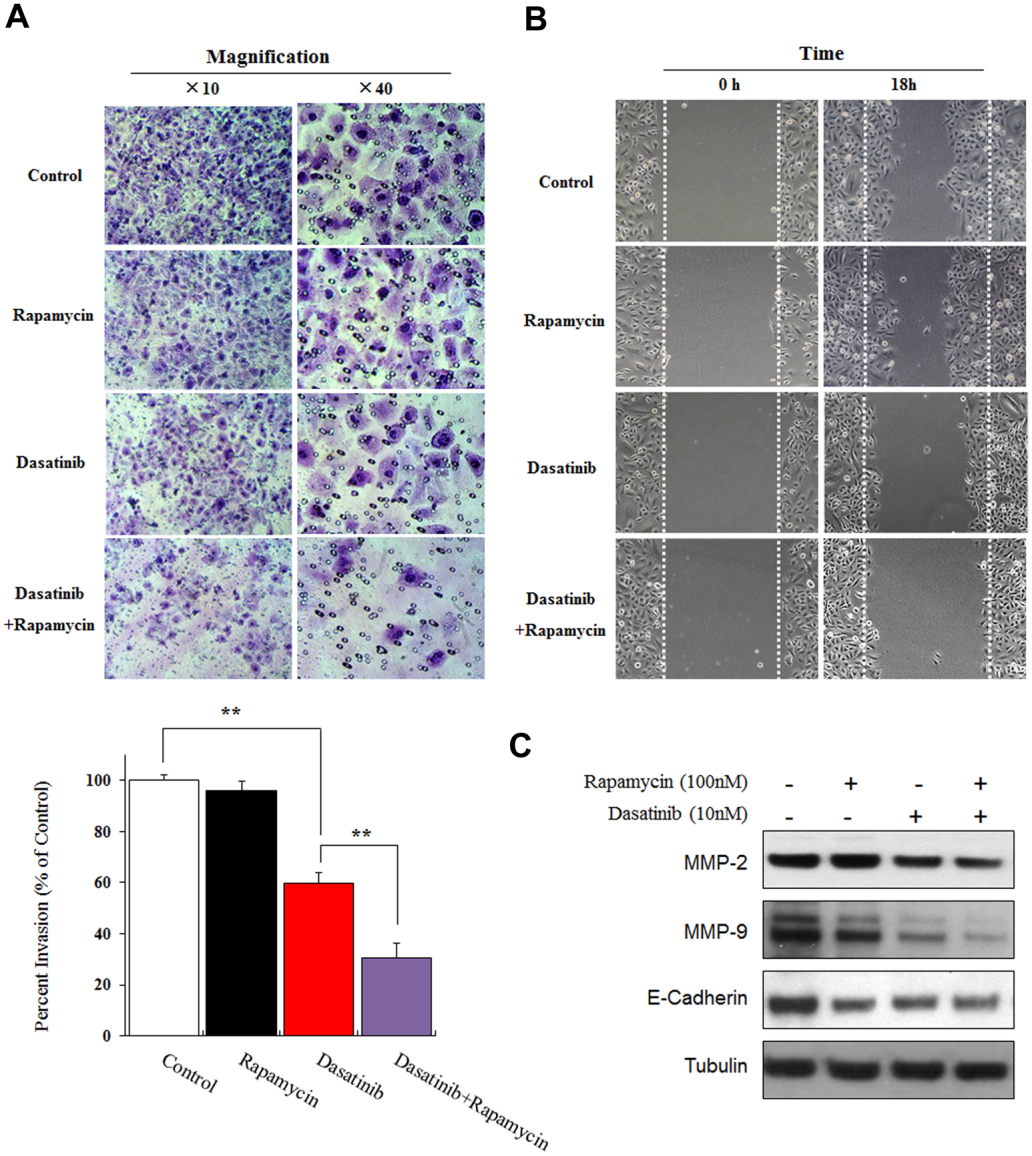
Rapamycin enhanced the inhibiting effect of Dasatinib on invasion and migration in A549 cells. (A) Representative images (upper panel) and quantification (lower panel) of invading cells. As detailed by “cell invasion assay” in the “*Methods*”, A549 cells were treated with DMSO (0.1%), Dasatinib (10 nM) alone or in combination with Rapamycin (100 nM) for 24 h. The numbers of invading cells were counted under microscope and the data was presented as mean ± SD from three independent experiments, * p < 0.05, ** p < 0.01. (B) Representative images of wound healing assays in A549 cells that were treated with DMSO (0.1%), Dasatinib (10 nM) or Rapamycin (100 nM) for 18 h. (C) The expression of MMP-2/9 and E-cadherin determined by western blotting in A549 cells that were treated with DMSO (0.1%), Dasatinib (10 nM) or Rapamycin (100 nM) for 24 h.

To further confirm the enhancing effect of Rapamycin on the anticancer activity of Dasatinib, we examined the invasion and migration of A549 cells under various treatments. As shown in Fig 5A, the invasive ability through Matrigel-coated filters of cells treated by Dasatinib was significantly decreased in comparison with the control cells. The co-treatment of Dasatinib with Rapamycin further decreased the invasion percent of A549 cells by nearly 50%, whereas Rapamycin alone did not exhibit clear suppression on cell invasion when compared to the control.

In addition, the effect of Dasatinib and Rapamycin on the migration ability of A549 cells was assessed by wound healing assay using physically wounded cells (Fig 5B). At 18 h after being scratched, the untreated A549 cells efficiently migrated into the incision, and the effect of Rapamycin alone on cell migration appeared limited. In contrast, the migration was obviously inhibited by the treatment with Dasatinib. Moreover, the migration was almost completely abolished in the cells treated with Dasatinib plus Rapamycin, suggesting the further-enhanced inhibition of cell migrating ability by the co-repression of Src and mTOR signaling.

It was generally demonstrated that PI3K/AKT signaling pathway can regulate metastasis in a variety of cancer cells [30], though the mechanism is still unclear by now. Matrix metalloproteinases MMP-2/9, as essential factors to cancer invasion and metastasis, mainly rely on PI3K/AKT/mTOR in A549 [31] and other carcinoma cells [32]. In addition, E-Cadherin plays an important role in controlling cell migration via PI3K-associated mechanism [33–35], and it was newly reported that loss of of E-Cadherin was associated with tumor metastasis in patients with NSCLC [36]. In our study, the expressions of MMP-2 and MMP-9 were suppressed by Dasatinib and further decreased by the combination with Rapamycin (Fig 5C). Similarly, significant decrease of E-Cadherin expression was also observed in A549 cells treated with the combination of Dasatinib and Rapamycin. Therefore, in addition to promoting the anti-proliferative effect of Dasatinib, Rapamycin was also capable of enhancing the inhibitory effect of Dasatinib on cell migration and invasion in A549 cells.

### Rapamycin enhanced the anti-cancer effect of Dasatinib in other NSCLC cells

**Fig 6:**
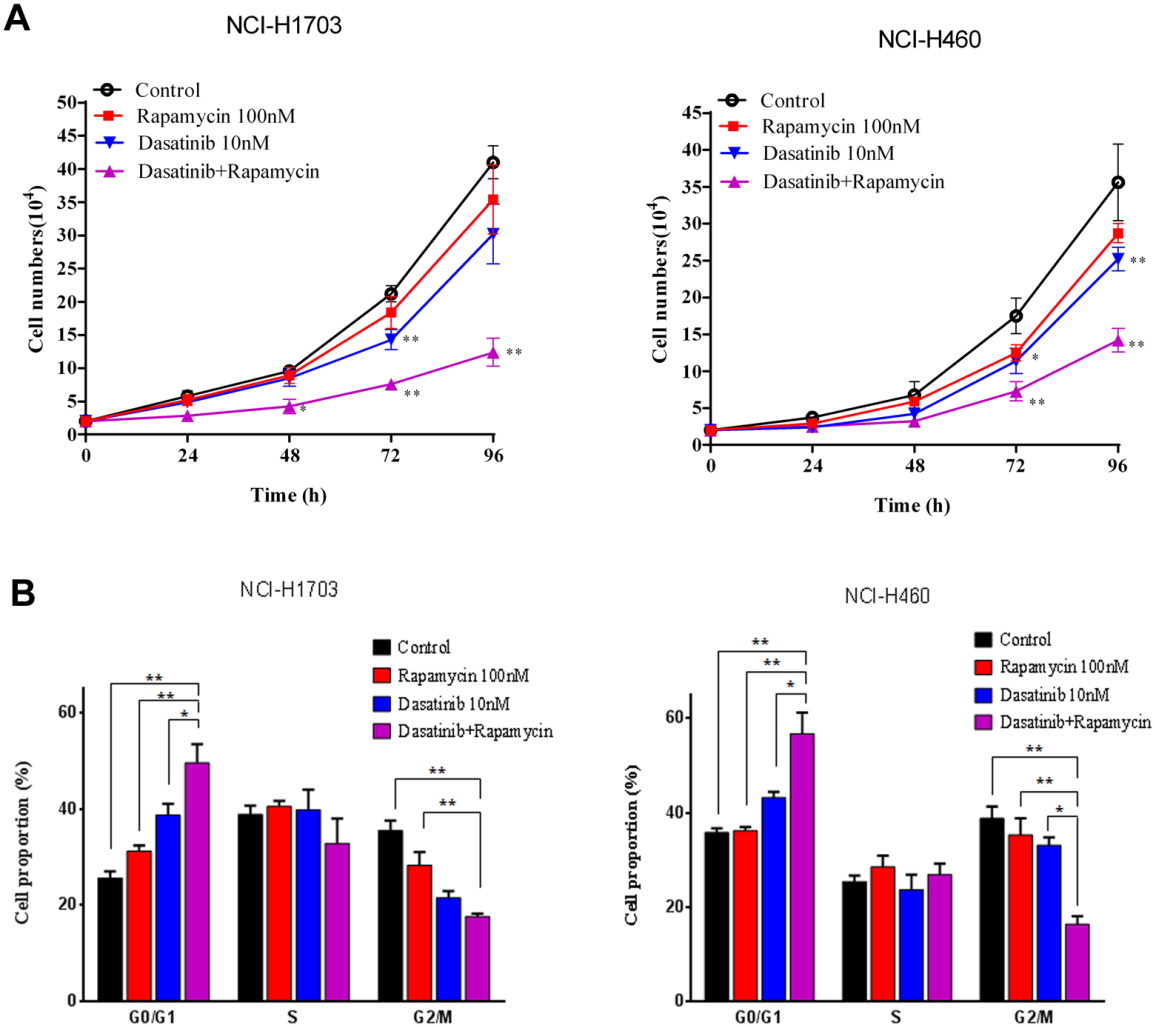
Rapamycin enhanced the inhibiting effect of Dasatinib on cell proliferation and cell cycle progression in other NSCLC cells. (A) Rapamycin enhanced Dasatinib-induced growth inhibition in NCI-H1706 and NCI-H460 cells. NCI-H1706 and NCI-H460 cells were treated with vehicle control (0.1% DMSO), Dasatinib (10 nM) with or without Rapamycin (100 nM). Cell numbers were measured at 0, 24, 48, 72, and 96 h after the treatment. (B) Effects of Dasatinib and Rapamycin on the cell cycle progression. NCI-H1706 and NCI-H460 cells were treated with Dasatinib (10 nM) or Rapamycin (100 nM) for 96 h and analyzed by flow cytometry after PI staining. Results are expressed as the average percentage of cells at G0/G1, S, and G2/M phase from three independent experiments. Columns, mean of three determinations; bars, SD. * p < 0.05, ** p < 0.01.

To validate the therapeutic significance of Rapamycin/Dasatinib combination found in A549 cells, we investigated the anti-cancer effects of the inhibitors in another two NSCLC cell lines, human lung squamous carcinoma cell line (NCI-H1703) and the human large-cell lung cancer cell line (NCI-H460). As shown in Fig 6A, the co-treatment with Rapamycin significantly enhanced Dasatinib-mediated growth inhibition in NCI-H1703 and NCI-H460 cells. The cell cycle analysis indicated that Rapamycin/Dasatinib combination remarkably increased the proportion of cells that arrest at G1 phase in both cell lines, in comparison with either Rapamycin or Dasatinib alone (Fig 6B). However, cell apoptosis was not significantly affected by the treatments in NCI-H1703 and NCI-H460 cells (S2 Fig), which was comparable to the results observed in A549 cells.

**Fig 7:**
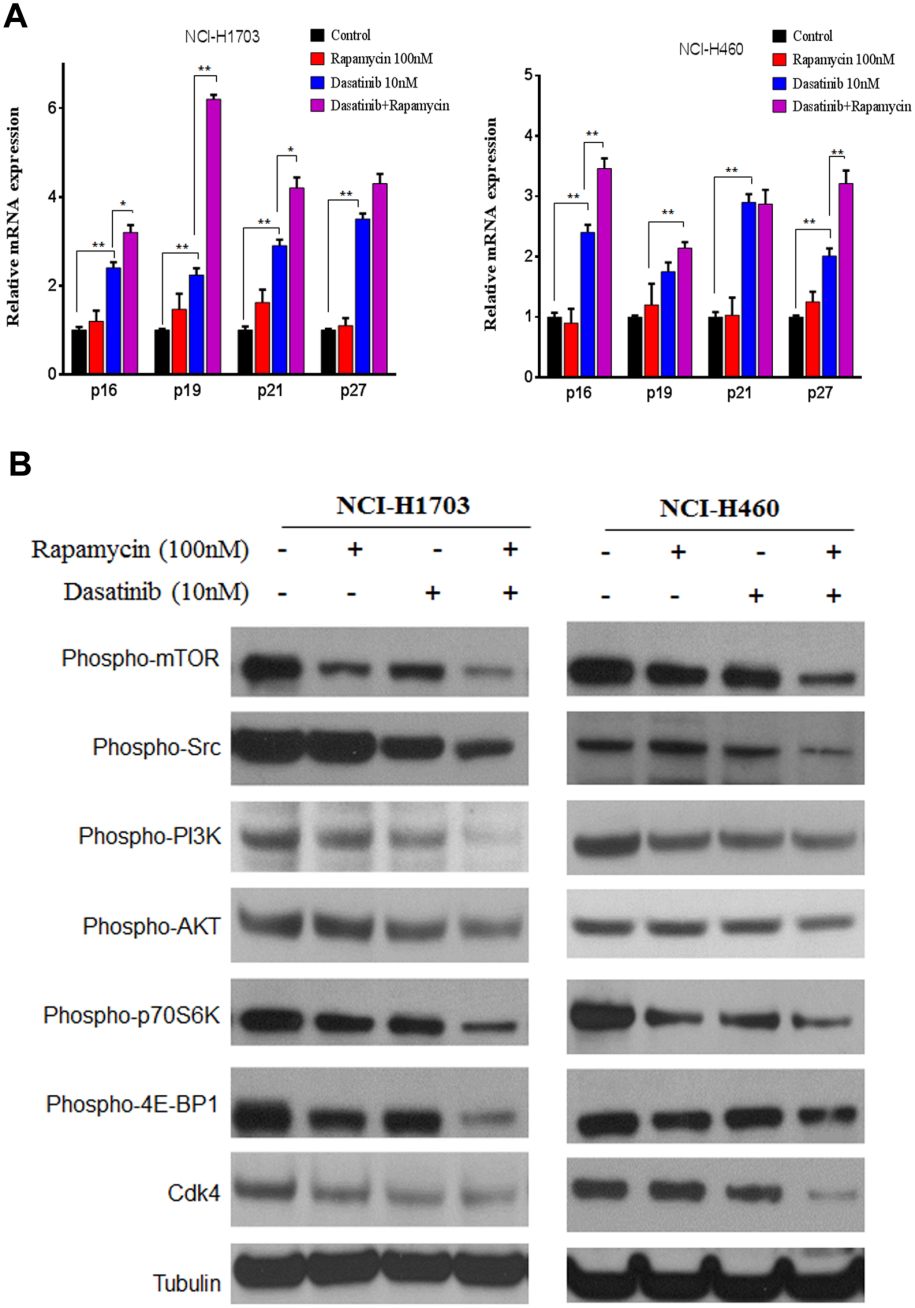
Dasatinib synergized with Rapamycin in up-regulating CDK inhibitors through Src/PI3K/Akt/mTOR pathway in other NSCLC cells. NCI-H1706 and NCI-H460 cells were treated with vehicle control (0.1% DMSO) or Dasatinib (10 nM) in the presence and absence of Rapamycin (100 nM) for 24 h. (A) Relative expression of CDK inhibitor proteins (p16, p19, p21 and p27) at mRNA level. Columns, mean of three determinations; bars, SD. * p < 0.05, ** p < 0.01. (B) The activation of Src, PI3K, AKT, and mTOR determined by western blotting. NCI-H1706 and NCI-H460 cells were treated with vehicle control (0.1% DMSO) or Dasatinib (10 nM) in the presence and absence of Rapamycin (100 nM) for 24 h. Homogenate proteins (20 μg) from the whole cell lysate was collected and used for western blotting.

To confirm whether the effects of Rapamycin and Dasatinib on cell proliferation and cell cycle progression were associated with CDK inhibitor proteins, we determined the expression of Cyclin A/D1/E as well as p16, p19, p21 and p27 at mRNA levels. Firstly, no significant alteration was found on the expression of cyclin A/D1/E and Cdc25A in both NSCLC cell lines under the treatments with Rapamycin or Dasatinib (S2 Fig). However, Rapamycin/Dasatinib combination significantly increased the transcription of p16, p19, p21 in NCI-H1706 cells, and p16, p19, p27 in NCI-H460 cells (Fig 7A).

In agreement with the connection between Src/PI3K activation and aberrant regulation of cell cycle in human lung cancer cells [13,14], we found that the endogenous phosphorylation levels of Src/PI3K were much higher in cancerous NSCLC cells than that in the normal human bronchial epithelial BEAS-2B cells (S3 Fig). Furthermore, analysis on Src and mTOR signaling in NCI-H1703 and NCI-H460 cells consistently demonstrated that the de-phosphorylation of PI3K/AKT and p70S6K/4E-BP1 were in parallel with the most significant inhibition of Src and mTOR induced by Dasatinib and Rapamycin, respectively (Fig 7B). In addition, the expression of Cdk4 was correspondingly decreased, and the most significant repression was observed in cells treated with Rapamycin and Dasatinib in combination. In summary, these results suggested that suppressing PI3K/AKT/mTOR pathway may be a general mechanism by which Rapamycin enhanced the anti-cancer effect of Dasatinib in NSCLC cells.

## Discussion

**Fig 8:**
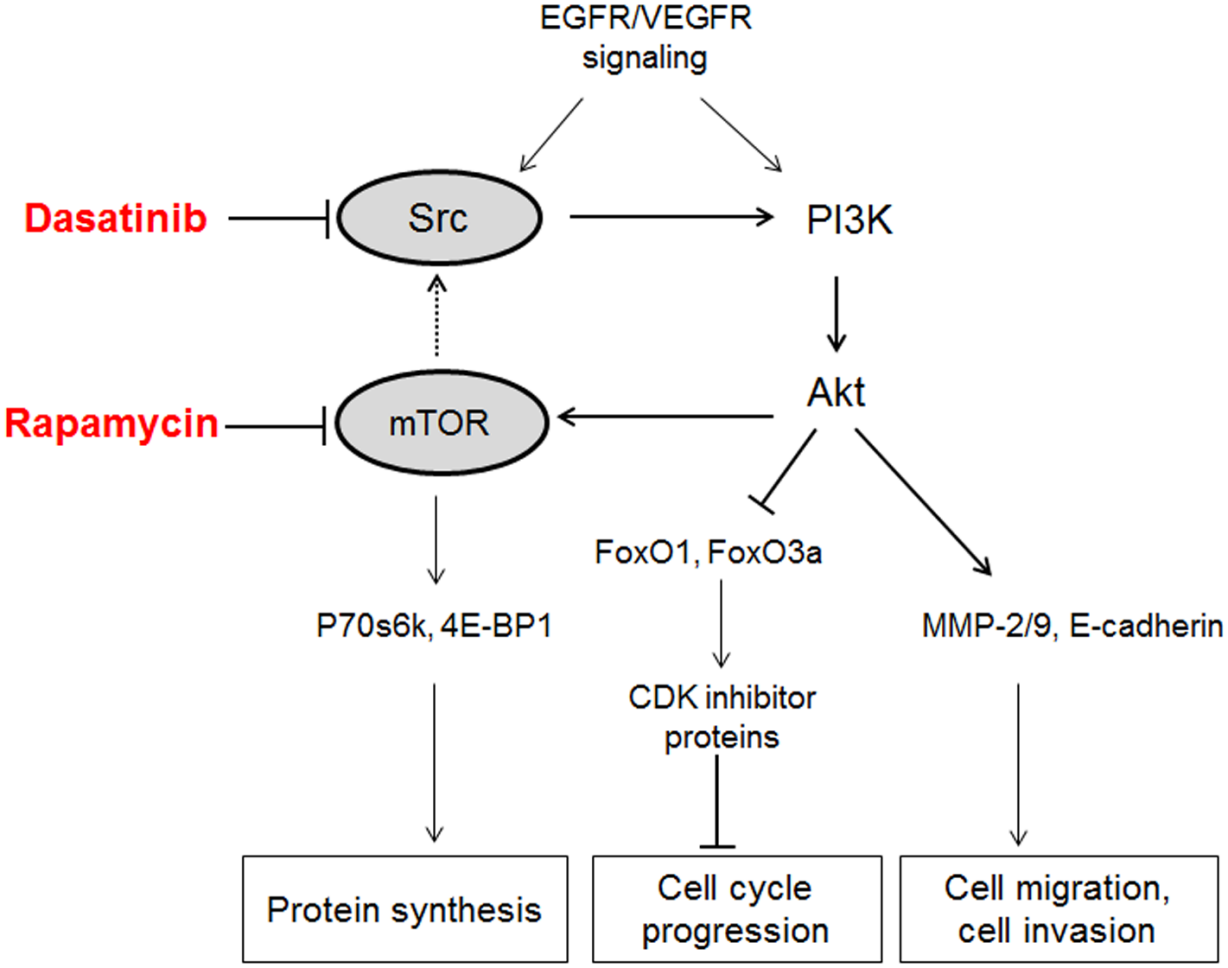
A schematic model for the anti-cancer function of Dasatinib and Rapamycin mediated by the Src-PI3K-Akt-mTOR pathway in NSCLC cells. Dasatinib-induced Src inhibition further suppressed the activation of mTOR via PI3K-Akt signaling pathway. The inhibition of mTOR by Rapamycin potentially facilitates Dasatinib-induced Src deactivation (dash line), thereby results in the enhanced up-regulation of CDK inhibitor proteins and cell cycle arrest via PI3K/AKT pathway. Meanwhile, the combination of Dasatinib with Rapamycin induces inhibition of cell migration and invasion through suppressing Src/PI3K/AKT signaling.

Although the combination of Dasatinib with other chemotherapeutic agents have shown superior efficacy for cancer treatment, mechanisms that lead to the enhanced sensitivity of Dasatinib are not clearly deciphered. In this study, we found Dasatinib-induced cell growth inhibition and cell cycle arrest were dramatically enhanced by the co-treatment with Rapamycin in multiple NSCLC cell lines. The mechanistic investigations suggested that the inhibition of Src and mTOR were potentiated by the combination of Rapamycin and Dasatinib via PI3K-AKT pathway, resulting in the up-regulation of CDK inhibitor proteins and down-regulation of Cdk4, as well as the repression of molecules governing protein synthesis, cell invasion and migration (Fig 8).

The function of Src as an oncogenic protein in cell cycle progression has been extensively investigated. It is well known that the cell cycle is negatively controlled by cyclin-dependent kinases (CDKs) inhibitor proteins; for example, p16 and p19 interact with cyclin D-CDK4/6, p21 and p27 interact with cyclin E-CDK2, jointly controlling cells to transit through the G1 phase to enter into the DNA synthesis S phase. Recent studies indicated that Src phosphorylation inversely correlated with p27 expression in a variety of carcinomas such as breast carcinoma [37], ovarian cancer [38], neoplastic esophagus cell lines [39], and mesothelioma cell lines [40]. Similar results were also observed from other CDK inhibitors [41,42]. In this study, we observed significant increase of p16, p19, p21, and p27 by Dasatinib which was further enhanced by the presence of Rapamycin (Fig 2B). Consistently, no clear changes were found in the expression of Cyclin A/D1/E, Cdc25A, and Cdk2/6, but the expression of Cdk4 was decreased. In addition, the cytosolic retention of Cdk4 which blocks the G1/S transition was inversely correlated with Src activation (Fig 3A). These results indicated that up-regulation of CDK inhibitor proteins and subsequent Cdk4 repression may be responsible for the cell cycle arrest and proliferation inhibition induced by Dasatinib alone or in combination with Rapamycin.

Since CDK inhibitor proteins can be transcriptionally regulated by FOXOs which has been indicated as downstream targets of PI3K/AKT [25,27], we speculated that the up-regulation of CDK inhibitors by Dasatinib and Rapamycin was probably attributed to the Src and PI3K/AKT pathway. As expected, the phosphorylation levels of FOXO1 and FOXO3a were markedly increased by the co-treatment when compared to that induced by either Src or mTOR inhibitor alone; moreover, the result was in parallel with the expressions of CDK inhibitors (Fig 2B) as well as de-activation of Src/PI3K/AKT and mTOR (Fig 3). Though it was previously reported that cell cycle progression can also be modulated by mTOR via p16, p21 or p27 [43,44], the best characterized process controlled by mTOR signaling is protein synthesis [16]. As an important translational regulator at the downstream of mTOR pathway, p70S6K and 4E-BP1 activation leads to an increase in mRNA biogenesis, as well as translational initiation and elongation, and thus, coordinates cell growth and survival [45]. Our data showed that mTOR inhibition by Rapamycin or siRNA induced significant decrease of p70S6K/4E-BP1 activation (Figs 3 and 4), which may contribute to synergizing the anticancer effect of Dasatinib.

On the other hand, the mTOR and p70S6K inhibition induced by Rapamycin was further enhanced by the co-treatment with Dasatinib, suggesting that Src played a crucial role in the regulation of mTOR signaling. It was reported that mTOR could be activated by Src signaling through PI3K/Akt pathway [22–24]. In agreement with previous studies, our result also indicated that consistent de-phosphorylation of mTOR/p70S6K was in line with the suppression of PI3K/Akt by Dasatinib (Figs 3 and 4). Similarly, other targets at downstream of PI3K/AKT pathway, MMP-2/9 and E-cadherin were also significantly suppressed, which agreed with the inhibition of cell invasion and migration by Dasatinib alone or in combination with Rapamycin in A549 cells (Fig 5). Together with the observation regarding enhanced repression of Src/PI3K/AKT by Rapamycin in multiple NSCLC cell lines, our results suggested that the synergistic anti-cancer effect of Dasatinib and Rapamycin was probably due to the inhibition of PI3K/Akt signaling transduction. Since a variety of assays in this study indicated that Rapamycin alone could also induce limited repression of Src/PI3K/Akt, it is possible that mTOR partially contributes to the regulation of Src signaling (Fig 8).

Although the impact of mTOR on Src has not yet confirmed, Src/PI3K signaling is not completely independent in the response to Dasatinib. The activation of Src and PI3K are associated with EGFR or VEGFR signaling, which are closely involved in multiple cellular functions in the oncogenesis including proliferation, differentiation, apoptosis, and angiogenesis [46,47]. And because of that, the combined use of SFK inhibitors like Dasatinib and EGFR inhibitors might also exert synergistic or additive effects in cancer treatments [48]. In summary, though the activation of Src may have pleiotropic effects that depend on the cell type and context, our results clearly demonstrated that Rapamycin markedly enhanced the anticancer effect of Dasatinib by facilitating cell cycle arrest and repressing cell proliferation, invasion as well as migration through Src/PI3K/AKT/mTOR pathway, implying promising therapeutic benefit of mTOR/Src dual inhibition for NSCLC treatment.

## Supporting Information

S1 Fig Effect of Rapamycin and Dasatinib on the expression of Cyclin A/D1/E and Cdc25A in A549 cells. A549 cells were treated with vehicle control (0.1% DMSO) or Dasatinib (10 nM) in the presence and absence of Rapamycin (100 nM) for 24 h. (A) Relative expression of Cyclin A/D1/E and Cdc25A at mRNA level. Columns, mean of three determinations; bars, SD. * p < 0.05, ** p < 0.01. (B) Expression of Cyclin A/D1/E and Cdc25A determined by western blotting.
(TIF)

S2 Fig Effects of Dasatinib and Rapamycin on the apoptosis and CDK inhibitor proteins expression in A549 cells. (A) Cells were treated with Dasatinib (10 nM) or Rapamycin (100 nM) for 96 h and the apoptotic rates were determined by flow cytometry with Annexin-V and PI staining. (B) Cells were treated with vehicle control (0.1% DMSO) or Dasatinib (10 nM) in the presence and absence of Rapamycin (100 nM) for 24 h. Relative expression of CDK inhibitor proteins (p16, p19, p21 and p27) at mRNA level. Columns, mean of three determinations; bars, SD.
(TIF)

S3 Fig Comparison of Src/PI3K/AKT and mTOR activation between BEAS-2B and NSCLC cells. Whole cell lysates were collected from different cell lines and homogenate proteins (20 μg) were used for western blotting. The phosphorylation levels of Src/PI3K/AKT and mTOR were much higher in cancerous NSCLC cells than that in the normal human bronchial epithelial BEAS-2B cells.
(TIF)

S1 Table Information of primers for Real-time quantitative PCR. (DOC)
