# Supplementary figures and images for: Rapamycin Enhances the Anti-Cancer Effect of Dasatinib by Suppressing Src/PI3K/mTOR Pathway in NSCLC Cells
Source: PLoS One. 2015 Jun 10;10(6):e0129663. doi: 10.1371/journal.pone.0129663 (PMC4465694; doi:10.1371/journal.pone.0129663)

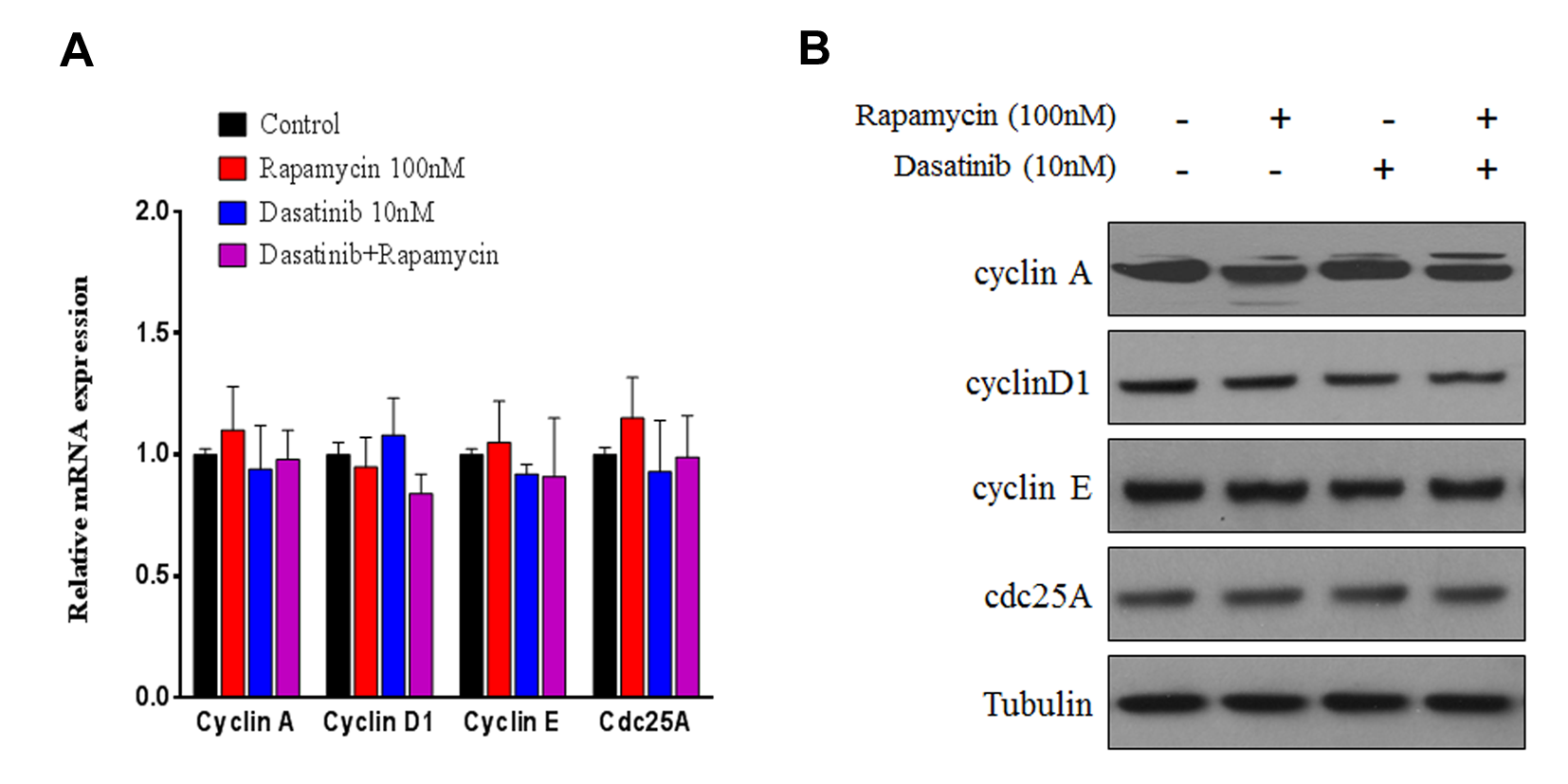

Supplement: S1 Fig — A549 cells were treated with vehicle control (0.1% DMSO) or Dasatinib (10 nM) in the presence and absence of Rapamycin (100 nM) for 24 h. (A) Relative expression of Cyclin A/D1/E and Cdc25A at mRNA level. Columns, mean of three determinations; bars, SD. * p < 0.05, ** p < 0.01. (B) Expression of Cyclin A/D1/E and Cdc25A determined by western blotting. (TIF) [file pone.0129663.s001.tif]

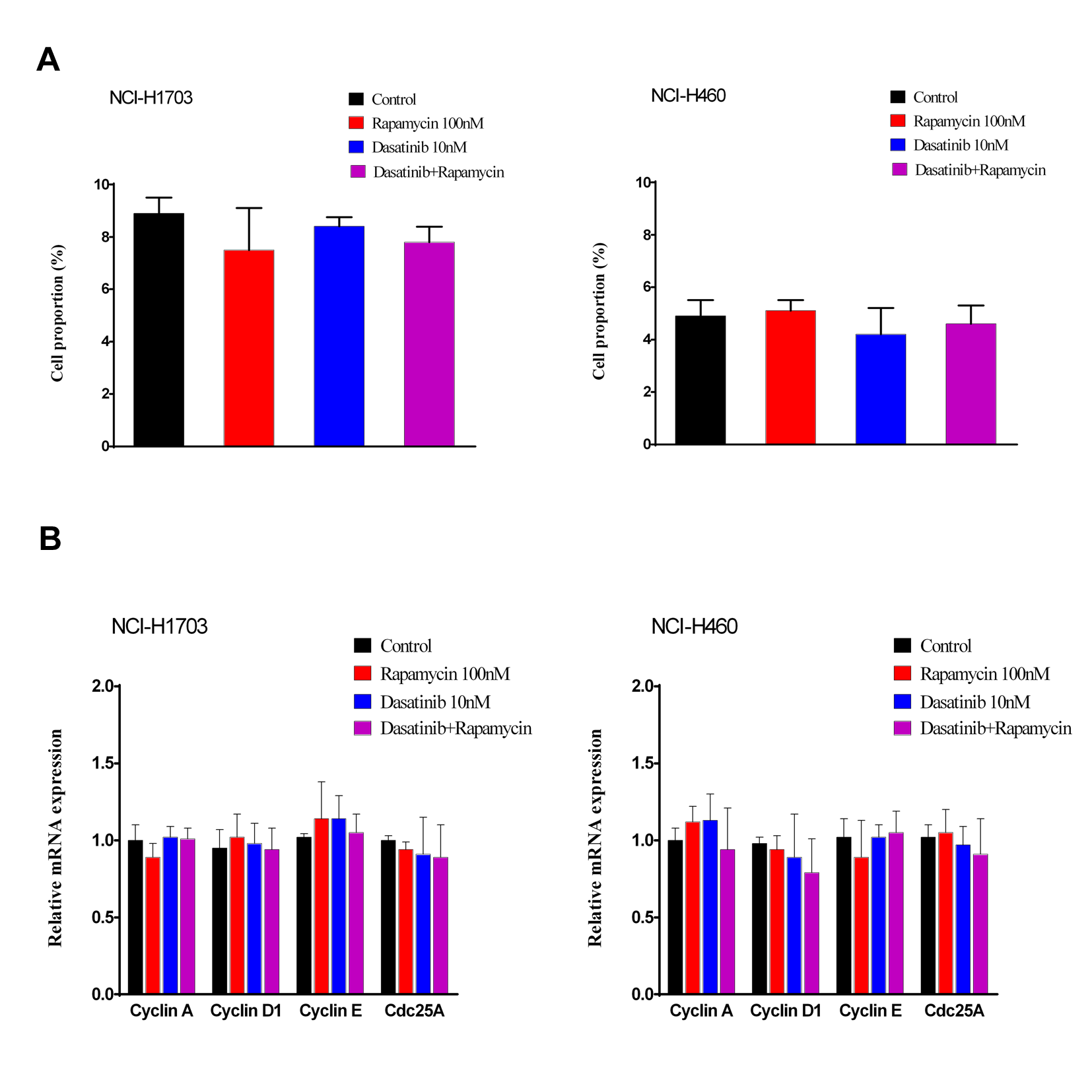

Supplement: S2 Fig — (A) Cells were treated with Dasatinib (10 nM) or Rapamycin (100 nM) for 96 h and the apoptotic rates were determined by flow cytometry with Annexin-V and PI staining. (B) Cells were treated with vehicle control (0.1% DMSO) or Dasatinib (10 nM) in the presence and absence of Rapamycin (100 nM) for 24 h. Relative expression of CDK inhibitor proteins (p16, p19, p21 and p27) at mRNA level. Columns, mean of three determinations; bars, SD. (TIF) [file pone.0129663.s002.tif]

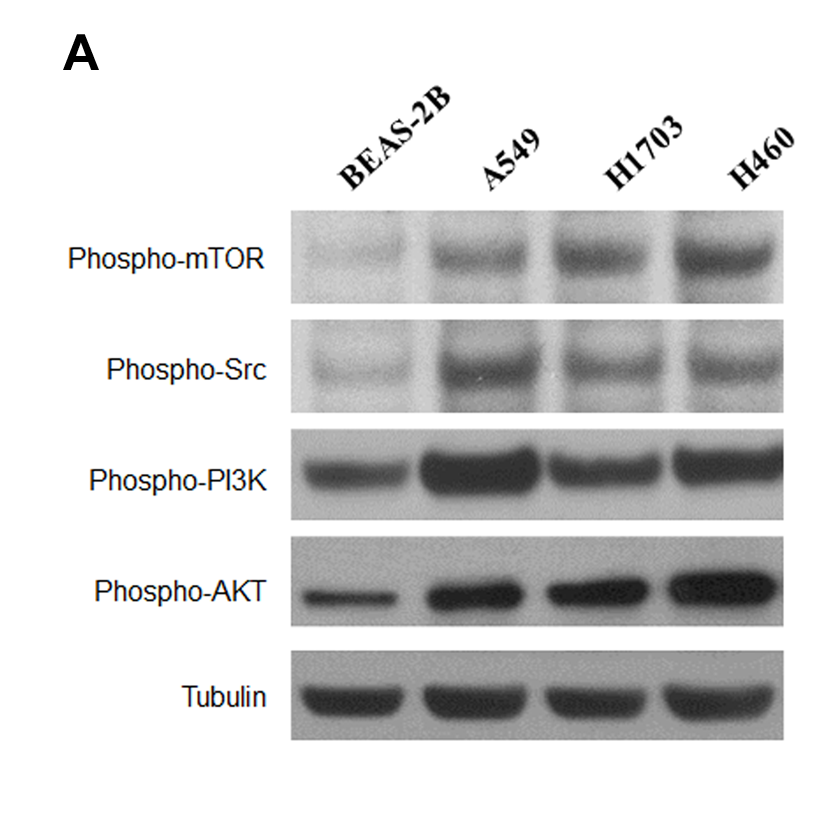

Supplement: S3 Fig — Whole cell lysates were collected from different cell lines and homogenate proteins (20 μg) were used for western blotting. The phosphorylation levels of Src/PI3K/AKT and mTOR were much higher in cancerous NSCLC cells than that in the normal human bronchial epithelial BEAS-2B cells. (TIF) [file pone.0129663.s003.tif]
