# Supplementary material for: Rapamycin Enhances the Anti-Cancer Effect of Dasatinib by Suppressing Src/PI3K/mTOR Pathway in NSCLC Cells
Source: PLoS One. 2015 Jun 10;10(6):e0129663. doi: 10.1371/journal.pone.0129663 (PMC4465694; doi:10.1371/journal.pone.0129663)
Supplement: S1 Table — (DOC) [file pone.0129663.s004.doc]

S1 Table Information of primers for Real-time quantitative PCR

| **Proteins** | **Gene Name** | **Forward (5’-3’)** | **Reverse (5’-3’)** |
| --- | --- | --- | --- |
| *p16* | CDKN2A | *GGGTTTCGCCCAACGCCCCGA* | *TGCAGCACCACCAGCGTGTCC* |
| *p19* | CDKN2D | *GTTTTCTTGGTGAAGTTCGTGC* | *TCATCACCTGGTCCAGGATTC* |
| *p21* | CDKN1A | *ACCTCTCAGGGCCGAAAAC* | *TAGGGCTTCCTCTTGGAGAA* |
| *p27* | CDKN1B | *CAGAGGACACACACTTGGTAGA* | *TCTTTTGTTTTGAGGAGAGGAA* |
| *Cyclin A* | CCNA1 | *ACATGGATGAACTAGAGCAGGG* | *GAGTGTGCCGGTGTCTACTT* |
| *Cyclin D1* | CCND1 | *GCTGCGAAGTGGAAACCATC* | *CCTCCTTCTGCACACATTTGAA* |
| *Cyclin E* | CCNE1 | *TCAAGACGAAGTAGCCGTTTAC* | *TGACATCCTGGGTAGTTTTCCTC* |
| *Cdc25A* | CDC25A | *GTGAAGGCGCTATTTGGCG* | *TGGTTGCTCATAATCACTGCC* |
| *18s RNA* | RNA18S5 | *GAAACGGCTACCACATCC* | *CACCAGACTTGCCCTCCA* |
